# A theoretical study of CO adsorption on Cu(211) surface with coverage effects

**DOI:** 10.55730/1300-0527.3427

**Published:** 2022-04-08

**Authors:** M. Oluş ÖZBEK

**Affiliations:** Department of Chemical Engineering, Gebze Technical University, Kocaeli, Turkey

**Keywords:** CO adsorption, Cu(211), DFT, adsorption energy, vibration, work function

## Abstract

CO adsorption on the Cu(211) surface was investigated using energy, geometry and vibrational data, which were produced through periodic DFT computations. Adsorption on the (111) terrace, as well as the previously reported top and bridge sites of the step-edges, was studied at 0.25, 0.33, 0.50, 0.66, 0.75, and 1.00 monolayer (ML) CO coverage. Results showed that up to and including 0.50 ML, on-top or bridge adsorption is preferred on the step-edges. When 0.50 ML is exceeded, top-bridge alternating patterns become feasible on the step edges along with possible shifts towards the terrace. Several feasible patterns were identified at 0.66, 0.75, and 1.00 ML. Like step-edge adsorptions, alternating patterns on the terrace sites were found feasible at higher coverages. For all the studied cases, highest adsorption energies were found for the step-edge positions. In general, coordination number had a stronger effect than coverage on the calculated properties.

## 1. Introduction

CO adsorption on the metallic copper or copper based catalysts is the initial step for the industrially applied heterogeneous catalytic reactions such as methanol synthesis [1,2] and water-gas shift reaction [1,3]. Vicinal surfaces are often found to enhance catalytic activity [4,5] and recent reports emphasize the catalytic importance of CO/Cu(211) interactions [1]. Nonetheless, available data is rather recent and limited for the CO interactions with stepped copper surfaces.

At very low coverage, CO appears to adsorb on the Cu(211) surface in a flat lying (parallel adsorbed) geometry [6,7], but this adsorption mode was not confirmed by the theoretical studies [8–10]. Up to 0.50 ML the ordered structures observed in STM images [11,12] in a p(2×1) pattern were reported to be CO molecules adsorbed on on-top or bridge sites of the step-edge Cu atoms [6–18]. At higher coverages, the alternating p(3×1) pattern observed at 0.66 ML and p(4×1) pattern at 0.75 ML [6,10–12] are more complicated and several configurations are possible [16]. For these alternating patterns on the step edges, CO adsorption in an alternating top-bridge configuration was suggested [10–12], whereas small nonperiodic CO islands appear to be singularly on-top adsorption [16]. Shifting of the CO molecules from the step-edges through the (111) terrace to reduce the repulsive forces is another possibility that was considered in a few works [8,9,16,19], but was not studied in detail.

For all the apparent simplicity of the CO/Cu(211) system, the energetic, geometric, and vibrational data is not complete. Especially, data for the thermodynamically unpreferred (111) terraces, the effect of coverage, and a deeper investigation of the alternating adsorption patterns is needed. Thus, this work presents a holistic data set produced through uniform and standardized periodic DFT computations for the CO/Cu(211) system. The results are presented below in three parts for the step-edge adsorption, the terrace adsorption, and the adsorption in alternating patterns with discussions.

## 2. Computational details

Quantum Espresso package [20] was used to perform the periodic DFT simulations. Norm conserving (NC) projector augmented wave (PAW) sets were used to describe the ionic core pseudopotential along with Perdew–Burke–Ernzerhof (PBE) functional for the exchange-correlation energy.

The Cu crystal structure was previously optimized with a lattice parameter of 3.643 Å [21]. P(1×1), p(1×2), p(1×3) and p(1×4) Cu(211) surface slabs were prepared with 5 atomic layers (6 layers in step edges) and 15 Å vacuum heights. During the simulations, the bottom 2 layers were kept frozen to represent the bulk, whereas the remaining top layers and the interacting CO molecule(s) were relaxed. The Brillouin zone sampling was done using automatically generated Monkhorst–Pack k-points with 4×12×1, 4×6×1, 4×4×1 and 4×3×1 meshes for the respective p(1×1), p(1×2), p(1×3) and p(1×4) slabs. The cut-off energies used were 50 Ry and 350 Ry for the wave functions and the charge densities, respectively. The k-points mesh, and cut-off energies were selected such that the calculated CO adsorption energies are within 1 kJ/mol confidence interval (see Figure S1). All the presented results were obtained by relaxing the structures until the net force acting on the ions was F_net_ < 0.001 Ry/Bohr. Necessary dipole corrections due to the asymmetric usage of slabs were included in the simulations.

Gas phase CO molecule was modeled in a vacuum supercell with a single gamma point, where the periodic molecules were separated with a minimum of 10 Å vacuum distances in all Cartesian coordinates. The adsorption energies of the CO molecules were calculated as the difference between the DFT energies of the products (CO adsorbed structure) and the sum of the reactants (clean Cu(211) surface + CO_(g)_),


$$\Delta E_{ads}=E_{CO/Cu(211)}-(E_{CO_{\left(g\right)}}+E_{Cu(211)})$$

CO coverage (*θ_CO_*) was calculated with respect to the number of edge atoms of the slab, even though the molecule may be adsorbed on the terrace. For 1.00, 0.50, 0.33, and 0.25 ML a single CO molecule was adsorbed on the p(1×1), p(2×1), p(3×1) and p(4×1) slabs, respectively. A total of 1.00 ML was also modeled by adsorbing two CO molecules on the p(2×2) slab, which allowed the study of the alternating patterns (see Table S1). When more than one CO molecule was present in model, the average adsorption energy is reported. Similarly, average C-Cu bond distances were reported for the 2-fold and 3-fold adsorptions. The work functions (Φ) were calculated as the difference between the Fermi energy (E_F_) of the structure and the electrostatic potential (ψ) of the vacuum,


$$\varphi =\psi -E_{F}$$

The vibrational frequencies of adsorbed surface species were obtained by calculating the Hessian matrix. During the frequency computations surface ions were excluded based on the frozen phonon approximation as well as the symmetry operators. Vibrational modes were assigned by visualizing each computed frequency. The Cu-C stretching vibrations (S-mode) were reported for the systems where a single CO was present. When there was more than one CO molecule in the model, the coupling of the frustrated modes and corresponding frequencies prevented a clear assignment.

## 3. Results and discussion

**Figure 1:**
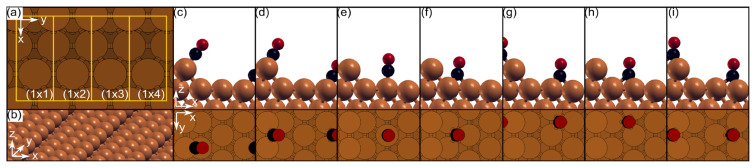
(a) Top views and the sizes of the studied Cu(211) slabs. (b) A perspective view of the Cu(211) surface that shows the step edges and the terraces. The remaining figures are the side and top views of the studied adsorption positions in the following order: (c) T1, (d) B, (e) T1, (f) F1, (g) F2, (h) H1, and (i) H2. (Cu: brown, C: black, O: red).

Figure 1 shows the studied p(1xn) slabs and the adsorption positions. Cu(211) surface consists of a 3 atoms wide (111) terrace and a (100) step. Following the existing literature [7–11,16,22,23] CO adsorption was studied on-top of the edge atom (T1) and in the bridging (B) site between two edge atoms. In addition, CO adsorption on the (111) terrace was studied for the on-top (T2), 3-fold fcc (F) and hcp (H) sites. Since two different sites exist for the 3-fold adsorptions, these were referred as F1, F2, H1, and H2. CO adsorption as it leans on the step side [7], bridging site on the (111) terrace and flat lying [7] geometries were also tested but stable adsorption modes could not be identified regardless of coverage.

### 3.1. CO adsorption on step edges

**Figure 2:**
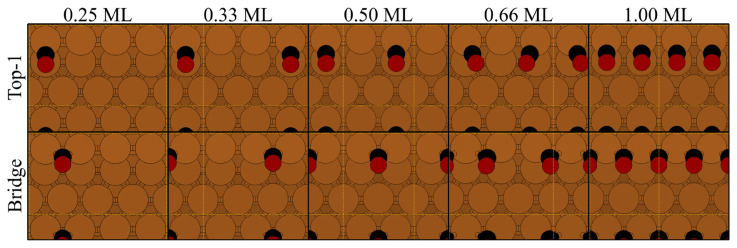
Top views of the CO adsorption on step-edge T1 and B positions. Feasible geometries for the monotonous T1 and B modes could not be obtained for 0.75 ML. (Cu: brown, C: black, O: red. Dashed lines represent the periodic boundary conditions.)

Table 1 shows the data obtained upon T1 and B adsorptions of the CO molecule, whose respective geometries can be seen in Figures 1 and 2. Up to 0.5 ML the adsorption energies, C-O and C-Cu distances of the T1 and B adsorption are not affected by the coverage. There is a consensus [7,10,11,16] that the monotonic structures observed up to 0.5 ML are the CO molecules occupying the on-top sites of the edge Cu atoms (denoted as T1). The structural data presented in Table 1 shows almost a perfect agreement with the previous reports. The tilting of the CO axis towards the lower terrace in T1 and B modes ( Figure 1 ) was reported experimentally [6,11,12], as well. The wide angles between the (111) terrace and the axis CO molecule axis are 123.85° and 114.34° for T1 and B modes, respectively. Once again, these angles are not affected by the coverage. At 0.75 ML, geometry relaxations produced only alternating patterns and will be discussed later.

Calculated adsorption energies of approximately 90 kJ/mol for Cu(211) are high compared to experimentally reported values of 38–70 kJ/mol of polycrystalline copper [24,25], and approximately 60 kJ/mol for Cu(211) [26]. However overestimated, the adsorption energies agree with the previous reports, where a similar level of detail was applied. In accordance with a previous study [10], T1 and B modes are separated only by 2~3 kJ/mol, thus leaving the site preference unconclusive at this point.

The high adsorption energies are most probably an artifact of the employed exchange correlation [18,27]. Although it has a very effect on the geometrical and vibrational results, it is known to strongly influence the adsorption energies [18]. When a correction is applied, these energies may reduce to the experimentally reported ranges, as well as making the energetic distinction between T1 and B modes more pronounced [10,28]. Several correction methods exist with their unique advantages and disadvantages, as discussed in [29–31]. However, within the purpose of this study, energy corrections were not employed, and are the subject of the following study. It should be noted that high adsorption energies alone do not render the results invalid since adsorption energies are not the solemn descriptors of the adsorption mechanism [31].

Along with the C-O and C-Cu distances, CO stretching frequencies are also in good agreement with the previous reports, bridge frequencies being lower than on-top values, as expected [7]. On the other hand, unlike the adsorption energies and the bond lengths C-O and C-Cu stretching frequencies change with the coverage and coordination number, making them a good descriptor for the adsorption mode when the adsorption energy is not a distinctive property [32]. Although the distance and the molecular vibration data show an almost perfect agreement with the reference values, the C-Cu (S-mode) vibrations were found almost approximately 100 cm^−1^ above compared the reference values. Similar frequency values were computed within this work, as well (for example, a 301 cm^−1^ exists for T1 adsorption in 0.33 ML), but a visual analysis show that these belong to the frustrated rotation and/or translation modes.

**Figure 3:**
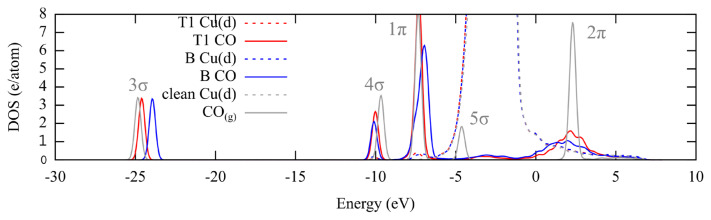
Projected density of states of CO adsorbed in T1 and B positions along with the d-bands of the top layer Cu atoms at 0.25 ML. Clean Cu(211) surface and the gas phase CO molecule are included as reference. The band positions are given with respect Fermi energy (Ef = 0 eV).

Although the differences are small, the changes in the C-O stretching frequencies without any apparent change in the adsorption energies and/or C-O bond distances point to a change in the electronic and bond structure of the Cu-C-O system. Blyholder model [33] states that the CO-metal bonding can be explained by 5σ (HOMO) donation to the metal surface followed by backdonation into 2π (LUMO). Further refinements to the model were added by Stroppa et al. [34]. The interaction of the 5σ with the surface s-states is attractive. Increasing coordination number increases the overlap between the metal d-bands and the 2π. Charge addition into the 2π (antibonding) orbital weakens the C-O bond, resulting in an increase in the bond length and decrease in the stretching frequency. Figure 3 shows that there is almost no change in the d-bands of the Cu surface. Upon adsorption 5σ broadens and shifts to a higher energy level (donation) and 2π interacts with the d-states resulting in a broadened hybrid structure (back-donation). Furthermore, 3σ and 1π bands of the bridge adsorption lye at a higher energy level, showing a less stable C-O bond, compared to the T1 adsorption. At this point, a charge analysis was used to determine the charge gained by the CO-p orbitals, which forms the majority of the 2π. At 0.25, 0.33 and 0.50 ML, the fractional occupancies of the CO-p orbitals increase by 0.32 for T1 and 0.49 for B modes, with respect to gas phase CO (Table S1). Although energies are similar for T1 and B adsorptions, there is a difference in electronic structure, which is reflected into the distances and the vibrations.

The work function of the clean Cu(211) surface was reported to be 4.46 eV [10]. In this work it was found to be a slightly higher as 4.49 eV. Increase in the work function with the coordination number and the coverage was expected [10], and the changes are large enough to be used as a descriptor of the adsorption modes. Furthermore, the 0.2 eV difference between the B and T1 at 0.66 ML agrees with the previous reports [10].

### 3.2. CO adsorption on (111) terrace

**Figure 4:**
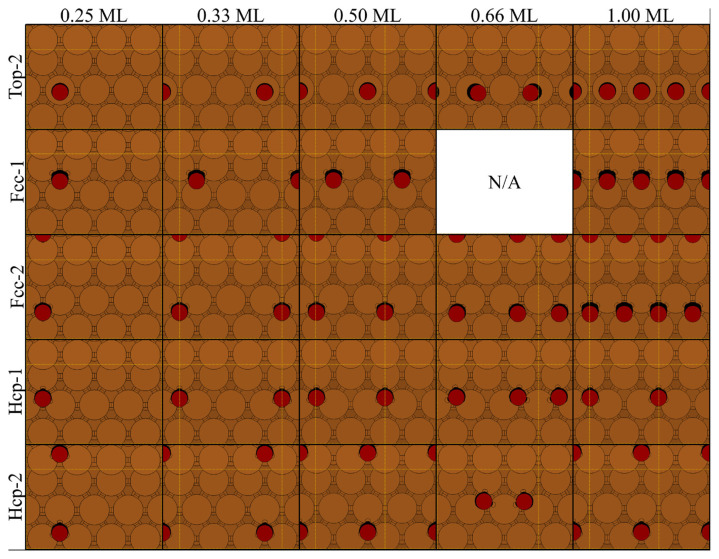
Top views of the CO adsorption on terrace sites at given coverages. Feasible monotonous adsorption modes could not be obtained for 0.75 ML, along with the F1 mode at 0.66 ML. (Cu: brown, C: black, O: red)

Table 2 gives the energy, geometry and vibrational data obtained for the CO adsorption on (111) terrace in on-top (T2), 3-fold fcc (F1 and F2) and hcp (H1 and H2) sites. Respective geometries can be seen in Figures 1 and 4. Stable adsorption modes for the bridge position on the terrace and F1 position for the 0.66 ML could not be identified.

To our best knowledge, previous reports do not present any data for the CO adsorption on the (111) terrace of the Cu(211) surface. Furthermore, comparison with the CO/Cu(111) system appears to be misleading since without any computational corrections the default CO adsorption are known to suggest the F1 site as the most feasible [18,29]. However, the results agree more with the experimental reports where CO on-top adoption is the preferred mode [29,35] and the T1 energies are closer to experimental reports for Cu(111) surface that are around 40–50 kJ/mol [31]. Here again the energy and distance data show little or no difference with the changing coverage, but the effect is more pronounced for the stretching frequencies.

A comparison of Tables 1 and 2 shows that the CO adsorption on the step-edges (T1 and B) is preferred at all coverages. However, compared to F1 and H1 modes that rest on the (111) plane, F2 and H2 positions near the step-edges appear to be more stable by approximately 20 kJ/mol and close to T1 and B scale. This can be attributed to the availability of the metal d-shells being more open to the adsorbing molecule due to the low coordination of the step-edge Cu atoms [31,36]. When the 0.5 ML is exceeded, once again the alternating structures become more feasible compared to monotonous adsorptions (such as T1+T1). This point will be discussed under the next heading.

The C-O and C-Cu bond lengths vary with the coordination number, rather than coverage. Bond lengths agree with the previously reported values for metallic copper surfaces [18,28,29]. Strong effect of the coordination number on the CO stretching frequencies can be seen here, as well. Although S-mode vibrations are lowered by the coordination number, the change is not as large compared to the frequencies and distances. Unfortunately, a clear comparison could not be made due to the lack of data for Cu(211) terrace adsorption. Nonetheless, based on two available data points [9,17] it can be commented that S-mode vibrations appear to be approximately 100 cm^−1^ higher, as in the case of T1 and B.

**Figure 5:**
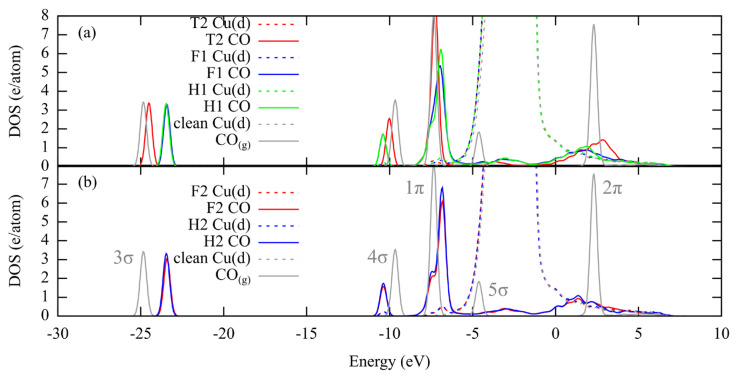
Projected density of states of CO adsorbed on terrace sites at 0.25 ML, along with the d-bands of the top layer Cu atoms at. Clean Cu(211) surface and the gas phase CO molecule are included as reference. The band positions are given with respect Fermi energy (E_f_ = 0 eV).

When Figures 3 and 5 are compared, it can be seen that 3σ energy levels are similar for T1, B and T2 modes, whose adsorption energies are also similar. Change in the 5σ appears to be similar for all the compared modes. For the remaining 3-fold adsorption modes 3σ and 1π shift into higher energy levels. The high coordination preference for the (111) surface was previously explained with the higher density of the d_xy_ and d_yz_ orbitals at the hollow sites, which would favor backdonation [31]. Once again, the change in the fractional population of the CO-p orbitals upon 3-fold adsorption is approximately 0.30 higher compared to T1 and T2, and approximately 0.15 higher (Table S1) compared to B modes, pointing to the increase in backdonation with the coordination number.

For the terrace adsorptions, the effect of the coordination number on the work function can be seen more clearly. The work function increases with the coverage as well as the coordination number, where the latter is more dominant.

### 3.3. CO adsorption in alternating patterns

**Figure 6:**
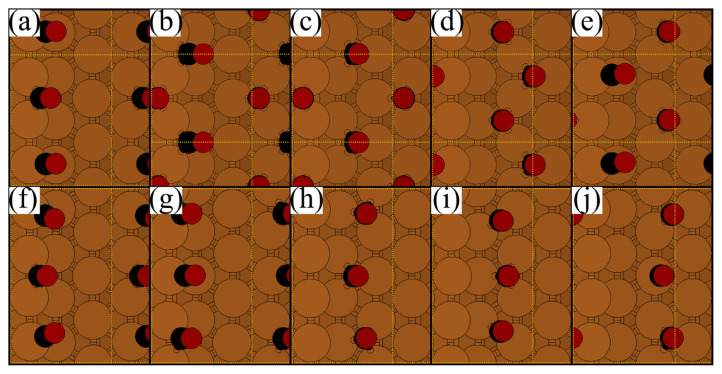
Top views of alternating patterns (a) T1-B at 0.66 ML, (b) B-H2 at 1.00 ML, (c) F1-H2 at 1.00 ML, (d) H1-F2 at 1.00 ML, (e) T1-F2 at 1.00 ML, (f) T1-B-T1 at 0.75 ML, (g) B-T1-B at 0.75 ML (h) H1-F1-H1 at 0.75 ML, (i) F1-H1-F1 at 0.75 ML, (j) F2-T2-F2 at 0.75 ML, (Cu: brown, C: black, O: red).

When the 0.50 ML is exceeded, CO molecules shift to adjacent positions to lower the lateral forces and the total energy, resulting in alternating patterns [7,10–12]. Although this phenomenon was reported for step-edges, a similar behavior is also true for the terrace adsorptions in this work. Table 3 gives the data obtained for the CO adsorption in alternating patterns at various coverages. Figure 6 shows the top views of these structures. A decrease in adsorption energies with increasing coverage was observed.

As the surface concentration increases, the first alternating pattern is observed at 0.66 ML in the form of T1-B adsorption [7,10,11,16]. The adsorption energy of alternating T1-B mode shows that it is slightly more stable by approximately 5 kJ/mol compared to monotonous T1 and B modes. It can be speculated that this small energy difference may not be enough to clear the ambiguity of the adsorption mode at 0.66 ML. At 0.75 ML, adsorption energies of the T1-B-T1 and B-T1-B alternating patterns are in 90 kJ/mol range. However, monotonous adsorption modes could not be obtained, and thus, cannot be compared. At 1.00 ML, T1-B type alternating pattern could not be obtained. Instead, second CO molecule shifts towards the F1 position, giving a T1-F1 type pattern. This structure is approximately 30 kJ/mol more stable compared to T1 adsorption. The preference of T1-F1 pattern instead of T1-B can easily be explained by the repulsion between two CO molecules at 1.00 ML. At 1.00 ML the repulsive force caused by the proximity of the two CO molecules in the would-be T1-B pattern (approximately 1.30 Å, which is the half of the C-C distance) is reduced by increasing this distance (approximately 4.00 Å) in T1-F1 mode.

Similarly, B-T1-B and T1-B-T1 patterns are favored due to the proximity of the singular T1 and B adsorptions. Although singular T1 adsorption was reported for small non-periodic CO islands at 0.66 ML [16], our findings are in line with the studies, where periodic CO adsorption was studied [7,10].

**Figure 7:**
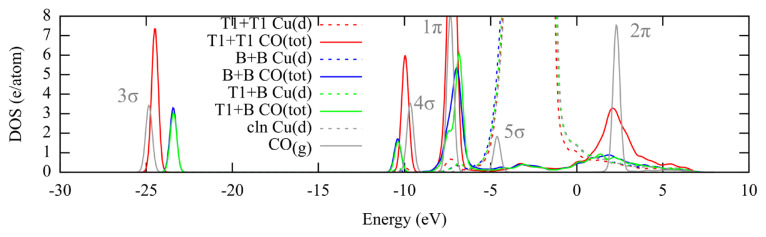
Projected density of states for the CO molecules adsorbed in T1+T1, B+B and T1+B modes at 0.66 ML, along with the d-bands of the top layer Cu atoms. Clean Cu(211) surface and the gas phase CO molecule are included as reference. The band positions are given with respect Fermi energy (E_f_ = 0 eV).

Figure 7 compares the band structures of the monotonous T1 and B adsorptions with that of alternating T1+B at 0.66 ML. There is a slight upshifting of all the bands of T1+B mode, which points to the weakening of the CO bond. When the 2π fractional donations are compared, the values are T1:0.34, B:0.47 (units in partial charges, electrons), T1+B: 0.40 (Table S3). The value for the T1+B mode is in the arithmetic average and can be attributed to the unchanging natures of the T1 and B modes. However, the upshifting of the orbitals that show a weakening of the C-O bonds can explain the stronger adsorption of the alternating structure.

Although the vibrational modes become coupled in the presence of multiple CO molecules, frequency values could be assigned based on the data given in Tables 1 and 2. Once again, the frequency values are distinctive enough to identify each adsorption mode. Furthermore, coordination number has a much stronger effect compared to coverage. A similar effect is also true for the work function. Although work function shows an increasing trend with coverage, a larger change is observed for the cases with higher coordination numbers.

## 4. Conclusion

Periodic DFT computations were carried out to investigate the CO/Cu(211) adsorption system through energy, geometry, and vibrational data. Along with the previously reported top (T1) and bridge (B) sites of the step-edges, CO molecule(s) were also adsorbed on the top (T2), fcc and hcp sites of the (111) terrace at 0.25, 0.33, 0.50, 0.66, 0.75 and 1.00 ML surface coverage. The results of the study can be summarized as follows.

- Up to 0.66 ML coverage preferred adsorption sites are the step-edge on-top (T1) and bridge (B) positions. PBE energies cannot differentiate between T1 and B at this point.
- At 0.66 ML and beyond alternating patterns are more feasible for both step-edge and terrace sites.
- For all considered cases step-edge adsorptions are preferred. Even on the (111) terrace, CO molecules prefer to adsorb near the step-edge positions.
- Coordination number has a stronger effect on all the measured quantities compared to coverage.
- Frequencies, distances, and work function are good descriptors of the adsorption modes.

### S.1. K-point convergence

The k-points mesh to be used in the computations was determined by calculating the CO adsorption energy on the top position of the step-edge Cu atom (denoted as Top1 in the manuscript). For this purpose the p(1×4) Cu(211) slab was used. Figure S1 shows the change of the adsorption energy with the increasing density of the k-points mesh along the Cartesian x- and y-coordinates. At the k-point values of 4 × 3 × 1, the change in the adsorption energy becomes less than 1 kJ/mol (for both directions), which is taken as the accepted margin of error for this study.

### S.2. 1/1 ML and 2/2 ML

Computations for 1.00 ML was performed on both p(1×1) and p(1×2) slabs. On p(1×1) slab a single CO molecule was adsorbed (1/1 ML), whereas on p(2×1) slab two CO molecules were adsorbed (2/2 ML). Table S1 shows the data obtained for both cases.

### S.3. Löwdin charge analysis

Löwdin charge analysis was carried out for the studied structures. Tables S2 and S3 show the charge gained by the CO-p orbitals (upon adsorption) with respect to gas phase CO (see main text for details).

Figure S1 Change of CO adsorption energy with the k-points used in the computations. The red line (4xn) shows the k-points used in Cartesian y-direction and the blue line (nx2) shows that of x-direction.

**Table S1:** Data obtained for 1/1 ML and 2/2 ML CO adsorption.

θ _CO_	Site	E _ads_ (kJ/mol)	|C-O| (Å)	|C-Cu| (Å)	ΔΦ (eV)	ν _C-O_ (cm ^−1^)
1/1 ML	T1	−61	1.153	1.850	0.30	2077
	T2	−37	1.155	1.863	0.23	2047
	B	−62	1.167	1.964	0.62	1964
	Fcc 1	−18	1.179	2.136	0.47	1846
	Fcc 2	−47	1.177	2.047	0.62	1874
2/2 ML	T1	−61	1.1531.153	1.849 1.849	0.30	1995 2078
	T2	−37	1.1551.154	1.860 1.860	0.24	1976 2047
	B	−62	1.1671.167	1.963 1.963	0.62	1892 1978
	Fcc 1	−18	1.179 1.179	2.054 2.054	0.48	1774 1850
	Fcc 2	−47	1.177 1.177	2.045 2.050	0.63	1788 1876

**Table S2:** Charge difference on the CO-p orbitals (w.r.t. CO_(g)_) for the given adsorption positions and surface coverage.

	Top 1	Top 2	Bridge	Fcc 1	Fcc 2	Hcp 1	Hcp 2
1.00 ML	−0.38	−0.40	−0.56	−0.64	−0.66	NA	NA
0.66 ML	−0.34	−0.36	−0.47	NA	−0.61	−0.61	−0.62
0.50 ML	−0.32	−0.35	−0.48	−0.63	−0.64	−0.62	−0.63
0.33 ML	−0.32	−0.34	−0.49	−0.64	−0.63	−0.62	−0.64
0.25 ML	−0.32	−0.34	−0.49	−0.63	−0.63	−0.62	−0.64

**Table S3:** Charge difference on the CO-p orbitals (w.r.t. CO_(g)_) for the given alternating adsorptions and surface coverage.

θ _CO_	Site	Δ
0.66 ML	T1+B	−0.40
0.75 ML	B+T1+B	−0.43
	T1+B+T1	−0.39
	F2+T2+F2	−0.53
	H1+F1+H1	−0.62
	F1+H2+F1	−0.57
1.00 ML	T1+F2	−0.48
	B+H2	−0.56
	F1+H2	−0.62
	H1+F2	−0.63

## Figures and Tables

**Table 1:** Adsorption energy (kJ/mol), C-O and C-Cu bonds distances (Å), change in work function (eV), C-O and Cu-C stretching frequencies (cm^−1^) for the studied coverages (ML) at step-edge top (T1) and bridge (B) sites.

θ_CO_	Site	E_ads_	|C-O|	|C-Cu|	ΔΦ	ν_C-O_	ν_C-Cu_
0.25	T1	−92	1.152	1.838	0.03	2045	413
	B	−95	1.169	1.970^b^	0.17	1906	336
0.33	T1	−92 −93[10] −66[10] −69[10] −89[28] −60[28]	1.152 1.154[10] 1.16[28]	1.839 1.85[10]	0.04	2050 2049[10] 2179[17] 2061[28]	431 333[10] 308[17] 343[9]
	B	−96 −95[10] −63[10] −57[10] −85[28] −43[28]	1.169^b^ 1.169[10] 1.18[28]	1.970^b^ 1.98[10]	0.22	1917 1925[10] 1902[28]	356 287[10]
0.50	T1	−92 −93[10] −66[10] −58	1.153 1.154[10]	1.839 1.84[10]	0.06	2055 2061[10] 2089[26]	439 342[10]
	B	−95 −95[10] −63[10]	1.167^b^ 1.168[10]	1.973^b^ 1.97[10]	0.30	1920 1938[10]	336 291[10]
0.66	T1	−91^a^ −91[10] −63[10]	1.155 1.154 1.156[10]	1.844 1.843 1.85[10]	0.14	2012 2056 2007[10] 2061[10]	407 404 341[10] 339[10]
	B	−90 a	1.165^b^ 1.165^b^	2.016^b^ 2.015^b^	0.35	1912 1965	448 263 334[10] 303[10]
1.00	T1	−61	1.153	1.850	0.30	2077	394
	B	−62	1.167^b^	1.964^b^	0.62	1964	340

a Average adsorption energy is reported when more than one CO molecule exists.

b Average bond length is reported for 2-fold and 3-fold adsorptions.

**Table 2:** Adsorption energy (kJ/mol), C-O and C-Cu bonds distances (Å), change in work function (eV), C-O and Cu-C stretching frequencies (cm^−1^), for the studied coverages (ML) at terrace top (T2), 3-fold fcc and hcp sites.

θ_CO_	Site	E_ads_	|C-O|	|C-Cu|	ΔΦ	ν_C-O_	ν_C-Cu_
0.25	T2	−67	1.154	1.850	0.01	2032	415
	F1	−56	1.186	2.051^b^	0.13	1799	401
	F2	−84	1.182	2.035^b^	0.17	1819	335
	H1	−72	1.182	2.067^b^	0.13	1825	388
	H2	−92	1.182	2.010^b^	0.20	1800	404.85
0.33	T2	−67	1.154	1.851	0.02	2027 2178[17]	416 309[17] 350[9]
	F1	−57	1.187	2.045^b^	0.17	1780	412
	F2	−84 −83[28] −43[28]	1.182 1.19[28]	2.037^b^	0.22	1827 1814[28]	378
	H1	−72	1.182	2.072^b^	0.17	1811	379
	H2	−93 −86[28] −40[28]	1.182 1.19[28]	2.006^b^	0.26	1819 1795	367
0.50	T2	−67	1.154	1.851	0.04	2025	400
	F1	−55	1.186	2.114^b^	0.25	1770	249
	F2	−82	1.182	2.032^b^	0.33	1816	346
	H1	−71	1.181	2.070^b^	0.25	1808	374
	H2	−91	1.181	2.058^b^	0.38	1821	315
0.66	T2	−66^a^	1.156 1.156	1.856 1.855	0.10	2001 2035	417 439
	F2	−78^a^	1.178 1.178	1.985^b^ 1.986^b^	0.41	1842 1885	389 348
	H1	−64^a^	1.179 1.179	2.139^b^ 2.026^b^	0.31	1808 1844	395 410
	H2	−64^a^	1.179 1.180	2.025^b^ 2.133^b^	0.31	1804 1841	308 426
1.00	T2	−37	1.155	1.863	0.23	2047	376
	F1	−18	1.179	2.136^b^	0.47	1846	308
	F2	−47	1.177	2.047^b^	0.62	1874	304

a Average adsorption energy is reported when more than one CO molecule was adsorbed.

b Average bond length is reported for 2-fold and 3-fold adsorptions.

**Table 3:** Adsorption energy (kJ/mol), C-O and C-Cu bonds distances (Å), change in work function (eV), C-O stretching frequencies (cm^−1^), for the studied coverages (ML) at terrace top (T2), 3-fold fcc and hcp sites.

θ_CO_	Site	E_ads_	|C-O|	|C-Cu|	ΔΦ	ν_C-O_
0.66	T1+B	−95 a −94[10] −65[10]	1.152 1.168 1.154[10] 1.170[10]	1.840 1.967 1.84[10] 1.97[10]	0.24	2059 1909 1908[10] 2057[10]
0.75	B+T1+B	−90 a	1.151 1.166 1.167	1.991 1.841 2.007	0.32	1907 1943 2057
	T1+B+T1	−92 a	1.153 1.153 1.169	1.845 1.846 1.965	0.25	1708 1774 2254
	F2+T2+F2	−74^a^	1.180 1.153 1.179	1.854 2.109 1.986	0.34	1827 1863 2030
	H1+F1+H1	−61^a^	1.179 1.186 1.179	2.049 2.127 2.034	0.35	1779 1844 1875
	F1+H2+F1	−61^a^	1.174 1.180 1.174	1.993 2.077 1.993	0.33	1845 1881 1904
1.00	T1+F1	−87^a^	1.184 1.150	1.848 2.042	0.35	1786 2068
	B+H2	−84^a^	1.182 1.165	1.985 2.040	0.53	1797 1945
	F1+H2	−72^a^	1.184 1.178	2.042 2.083	0.54	1782 1862
	H1+F2	−67^a^	1.179 1.181	2.044 2.028	0.53	1786 1854

a Average adsorption energy is reported when more than one CO molecule was adsorbed.

b Average bond length is reported for 2-fold and 3-fold adsorptions.
