# Supplementary figures and images for: A theoretical study of CO adsorption on Cu(211) surface with coverage effects
Source: Turk J Chem. 2022 Apr 8;46(4):1199–209. doi: 10.55730/1300-0527.3427 (PMC10395743; doi:10.55730/1300-0527.3427)

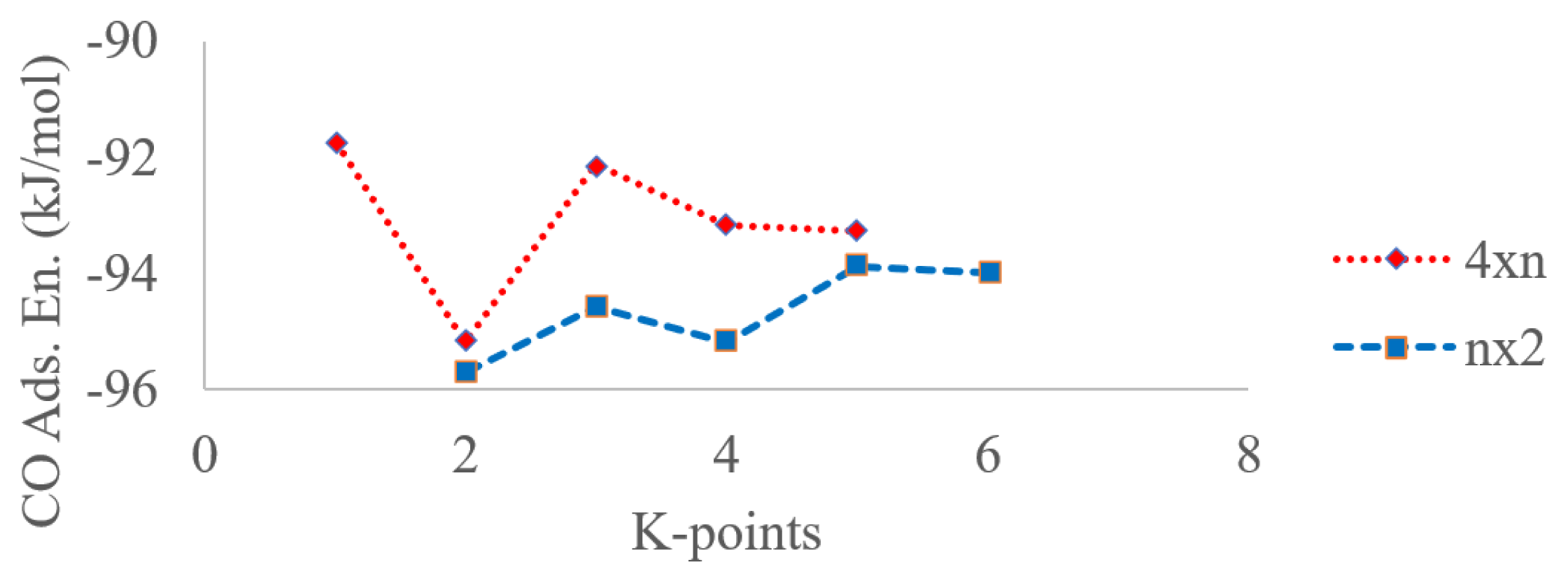

Supplement: Figure S1 — Change of CO adsorption energy with the k-points used in the computations. The red line (4xn) shows the k-points used in Cartesian y-direction and the blue line (nx2) shows that of x-direction. [file turkjchem-46-4-1199s1.tif]
